# Ubiquitin-proteasome system in *Plasmodium*: a potential antimalarial target to overcome resistance – a systematic review

**DOI:** 10.3389/fmed.2024.1441352

**Published:** 2024-10-21

**Authors:** Adriana F. Gonçalves, Ana Lima-Pinheiro, Pedro E. Ferreira

**Affiliations:** ^1^Life and Health Sciences Research Institute (ICVS), School of Medicine, University of Minho, Braga, Portugal; ^2^Life and Health Sciences Research Institute (ICVS), Biomaterials, Biodegradables and Biomimetics Research Group (3B's), PT Government Associate Laboratory, Braga, Portugal

**Keywords:** antimalarial resistance, artemisinin, ubiquitin-proteasome system, *plasmodium* spp., proteasome inhibitors

## Abstract

**Background:**

Malaria is a devasting parasitic disease that causes over half a million deaths every year. The necessity for prompt and thorough antimalarial drug discovery and development is accelerated by the rise in multidrug resistance and the lack of an effective vaccine. The *Plasmodium* spp. proteasome represents a prospective target for antimalarial treatment since several chemotherapy types have been shown to potently and selectively limit the growth of parasites. Combined with first-line artemisinin medicines, it creates synergy, even in the artemisinin-resistant parasites.

**Methods:**

PRISMA guidelines were used in the development of this systematic review. A literature search was performed in March 2024 in PubMed, Science Direct, and Scopus databases, with the following keywords: ((antimalarial resistance) AND (plasmodium OR malaria) AND (proteasome)) NOT (cancer [Title/Abstract]). Only articles with the susceptibility assessment were included.

**Results:**

Herein, 35 articles were included in the systematic review, which was divided into two subcategories: those that studied the UPS inhibitors, which accounted for 25 articles, and those that studied genetic modifications, including knockouts, knockdowns, and mutations, in the UPS toward antimalarial resistance, accounting for 16 articles. 6 articles included both subcategories. In total, 16 categories of inhibitors were analyzed, together with two knockdowns, one knockout, and 35 mutations.

**Conclusion:**

In this study, we reviewed the literature for available inhibitors and their respective susceptibility and ability to develop resistance toward *Plasmodium* spp. 26 s proteasome. The proteasome was highlighted as a potential antimalarial target and as an artemisinin partner drug. However, host toxicity and susceptibility to resistance appear as the main obstacle in the development of highly potent drugs, indicating a need for additional scrutiny during any further drug development efforts.

## 1 Introduction

Malaria accounted for 249 million cases and 608 thousand deaths in 2022, continuing to pose a significant health threat worldwide (1). It is caused by a eukaryotic protozoan parasite of the *Plasmodium* genus, transmitted through an infected *Anopheles* mosquito. Several *Plasmodium* species are known to infect and be transmitted to humans, being *P. falciparum* and *P. vivax* the major causative agents of human malaria. *P. falciparum is* the deadliest (2, 3).

The loss of first-line treatments due to resistance has been a persistent challenge in controlling this disease (4). Nowadays, artemisinin-based combination therapies (ACTs) have become the leading antimalarial, comprising short-lived ARTs (artemisinin and its derivatives) that rapidly reduce parasite biomass with a partner drug lasting longer for the clearance of remaining parasites (3). Nevertheless, artemisinin resistance has emerged in the Greater Mekong Subregion, and its spread into high-endemicity regions in Africa will have severe consequences (5). Unlike other antimalarials where resistance is defined as clinical treatment failure and reduced *in vitro* sensitivity, artemisinin resistance is characterized by the delay in parasite clearance time (6). This poorly correlates with the standard *in vitro* measures of susceptibility. Therefore, the ring-stage survival assay (RSA) was developed, given the need for a more reliable and standardized *in vitro* measure of delayed clearance that correlates with the *in vivo* resistance phenotype (7).

Artemisinin resistance is often linked to genetic variations in the *β*-propeller domain of the *P. falciparum* Kelch 13 (*Pf*K13) propeller protein. K13 is a crucial protein comprising BTB (Bric-a-brac, Tramtrack, and Broad complex) and KREP (Kelch-repeat propeller) domains, commonly encountered in ubiquitin ligase (E3) complexes responsible for directing substrate protein(s) toward ubiquitin-dependent degradation. Parasites carrying these mutations exhibit changes in the intraerythrocytic cell cycle, including lengthened ring and shortened trophozoite stages (8). Additionally, these parasites show molecular alterations such as increased expression of unfolded protein response pathways (9), reduced levels of ubiquitinated proteins (10, 11) and *Pf*K13 protein (12), and phosphorylation of parasite eukaryotic factor 2α (eIF2α) during the early intraerythrocytic stage. This phosphorylation event halts protein translation, leading to artemisinin-induced dormancy (9, 13, 14).

Artemisinin forms radicals that induce cellular damage by reacting with susceptible biomolecule groups, accumulating damaged and unfolded proteins. This generates a parasite’s stress response coping mechanism, leading to parasite death. The ubiquitin-proteasome system (UPS) is responsible for maintaining homeostasis and upholding protein quality control in the overwhelmed parasite. Considering this, the parasite’s modulation of the proteasome degradation pathway could contribute to artemisinin resistance (15).

The UPS has a posttranslational modification process in the proteins, named ubiquitination, which consists of the attachment of a polyubiquitin chain that is then recognized by the 26 s proteasome. The type of ubiquitination determines if the protein is recycled or degraded (16, 17). The *P. falciparum* 26 s proteasome and associated proteins have recently been characterized, revealing a few variations from studies in humans, which could be leveraged for therapeutic purposes (18). In mammalian cells, ubiquitin is bound to biomolecules progressively and under control. First, ubiquitin is processed proteolytically by ubiquitin-specific proteases (USPs) to reveal a diglycine motif, followed by an ATP-dependent ubiquitin binding to the cysteine active site of the ubiquitin-activating enzyme (E1). The ubiquitin-conjugating enzyme (E2) receives then the active ubiquitin. The ubiquitin ligase E3, which binds the substrate and the mediator via distinct structural motifs, uses the activated intermediate of E2-ubiquitin as a ubiquitin donor to the substrate. The same E3 enzyme or the ubiquitin chain-elongation enzyme (E4) can lengthen the ubiquitin chains that are then recognized by the proteasome. The deubiquitinases (DUBs) remove them from the substrate, whereas substrates are cleaved into short peptides and posteriorly broken down to amino acids by aminopeptidases (APPs). Finally, cytosolic DUBs recycle released polyubiquitin molecules for subsequent ubiquitylation (19, 20).

The 26 s proteasome is a barrel-shaped multi-subunit proteinase complex comprising a 20S core particle (CP) and a 19S regulatory particle (RP). The first one, CP, is responsible for proteolysis through peptidyl glutamyl-peptide hydrolytic (PGDH) (caspase-like), trypsin-like, and chymotrypsin-like activities, facilitated by its *β*-subunits (β1, β2 and β5, respectively). The RP is accountable for recognizing substrates, deubiquitinating, unfolding, and facilitating translocation (18, 19). These catalytically active subunits cleave after the carboxy-terminal side of hydrophobic, tryptic, and acidic residues, respectively, using an N-terminal threonine as the nucleophile. The two outside beta rings of the barrel, which are made up of α1–α7, block polypeptide access to the catalytic subunits, while the two inner beta rings, which are made up of β1–β7, contain these active sites. The 19S subunits control this gate opening of the 20S core. One key structural difference between human and plasmodial proteasomes is the unusually open β2 active site in *P. falciparum* proteasome (20, 21).

At least 18 protein subunits compose the 19S RP, structurally divided into two sub-complexes, lid and base. The first one comprises nine regulatory particle non-ATPase (rpn) subunits (3, 5, 6, 7, 8, 9, 11, 12, 15). The last one includes the other rpn and six regulatory particle ATPase (rpt) subunits, which are responsible for the protein unfolding and the CP gate opening. The insertion of the C-terminal of all rpt subunits, except rpt4, into the exterior pockets of the *α*-ring is required for this gate opening. Furthermore, they also maintain the stability of the 19S RP and 20S CP connection (19). The 19S RP utilizes two intrinsic ubiquitin receptor domains to recognize polyubiquitinated substrates: the ubiquitin-interacting motif (UIM) in the rpn10 subunit and the pleckstrin-like receptor for ubiquitin (Pru) domain in the rpn13 subunit. Additionally, there are many other proteins known as proteasome-interacting proteins (PIPs), which are extrinsic receptors that recruit and deliver to the proteasome ubiquitylated substrates. Among these, ubiquitin-binding proteins like Rad23 and Dsk2, which bind to the 19S via a ubiquitin-like (UBL) domain and associate with polyubiquitinated proteins via a ubiquitin-associated (UBA) domain, act as proteasome substrate shuttle factors. The 19S RP also associates with two DUBs, USP14/Ubp6 and UCH37, and a ubiquitin ligase, Hul5, which collaboratively modify ubiquitin chains on proteasomal substrates (18, 19).

Hence, the goal of this systematic review is to investigate and elucidate the prevalence and the role of the proteasome in the development of resistance to currently available antimalarials, aiming to contribute to the advancement of more effective treatments in the future. By collecting information about the proteasome and its inhibitors, we can help create more effective combination therapies and identify potential gene targets for new treatments.

## 2 Materials and methods

The Preferred Reporting Items for Systematic Reviews and Meta-analyses (PRISMA) guidelines were used in the development of the protocol for this systematic review (22). This review’s protocol was not registered before its submission.

### 2.1 Search strategies

A comprehensive literature search was conducted in March 2024 in the following electronic databases: PubMed, Science Direct, and Scopus, using the combination of keywords: ((antimalarial resistance) AND (plasmodium OR malaria) AND (proteasome)) NOT (cancer [Title/Abstract]).

### 2.2 Eligibility

The systematic review includes the following types of studies: (i) studies that assess the antimalarial susceptibility.

Studies were excluded when one of the following criteria were observed: (i) unpublished data (ii) non-original articles (reviews, protocols, encyclopedia, book chapters, systematic reviews); (iii) gray literature (conference abstracts, correspondence, thesis); (iv) articles written in languages other than English.

### 2.3 Study selection

Research articles identified from searches of the electronic databases were screened for eligibility based on their titles and abstracts. Duplicates and ineligible articles were removed. Full-length articles were then read to confirm for fulfillment of the inclusion criteria before data extraction. This selection of the articles was performed by two independent reviewers, as well as the data extraction from articles that fulfilled the inclusion criteria. Discrepancies were solved by mutual consent and/or after discussion and independent review from a third researcher, the supervisor.

### 2.4 Outcomes measured

The main outcomes focused on antimalarial susceptibility, measured as half-maximum inhibitory concentration (IC_50_) and/or RSA (this last one specifically for artemisinin studies), proteasome activity assays, and lines studied as well as mutations associated.

### 2.5 Data extraction, management and synthesis

The data were extracted from included studies and compiled in a Microsoft Excel spreadsheet, including the following information: author, year of publication, study design, species of *Plasmodium*, type of antimalarial studied, and outcomes measured. To manage the references of the articles, the Mendeley Desktop (Version 1.19.8 – Elsevier) bioinformatic tool was used.

### 2.6 Quality assessment

For quality assessment, an adaptation to *in vitro* experiments of the ARRIVE (Animal Research: Reporting of *in vivo* Experiments) guidelines was used (23), due to the lack of a scale to evaluate these experiments. A total of 14 criteria were assessed, including (1) study design, (2) inclusion and exclusion criteria, (3) outcome measures, (4) statistical methods, (5) description of species, (6) experimental procedures, (7) results, (8) abstract, (9) background, (10) objectives, (11) interpretation and/or scientific implications, (12) generalizability/translation, (13) data access and (14) declaration of interests. The potential range of the ARRIVE adapted quality score is 0–14. A score between 0 and 1 was attributed to each criterion and the mean score was calculated for each study. A global rating of good study was attributed to studies with a mean score higher than 12, moderate for a score between 8.5 and 12, and weak for a score lower than 8.5.

## 3 Results

### 3.1 Literature search results

**Figure 1:**
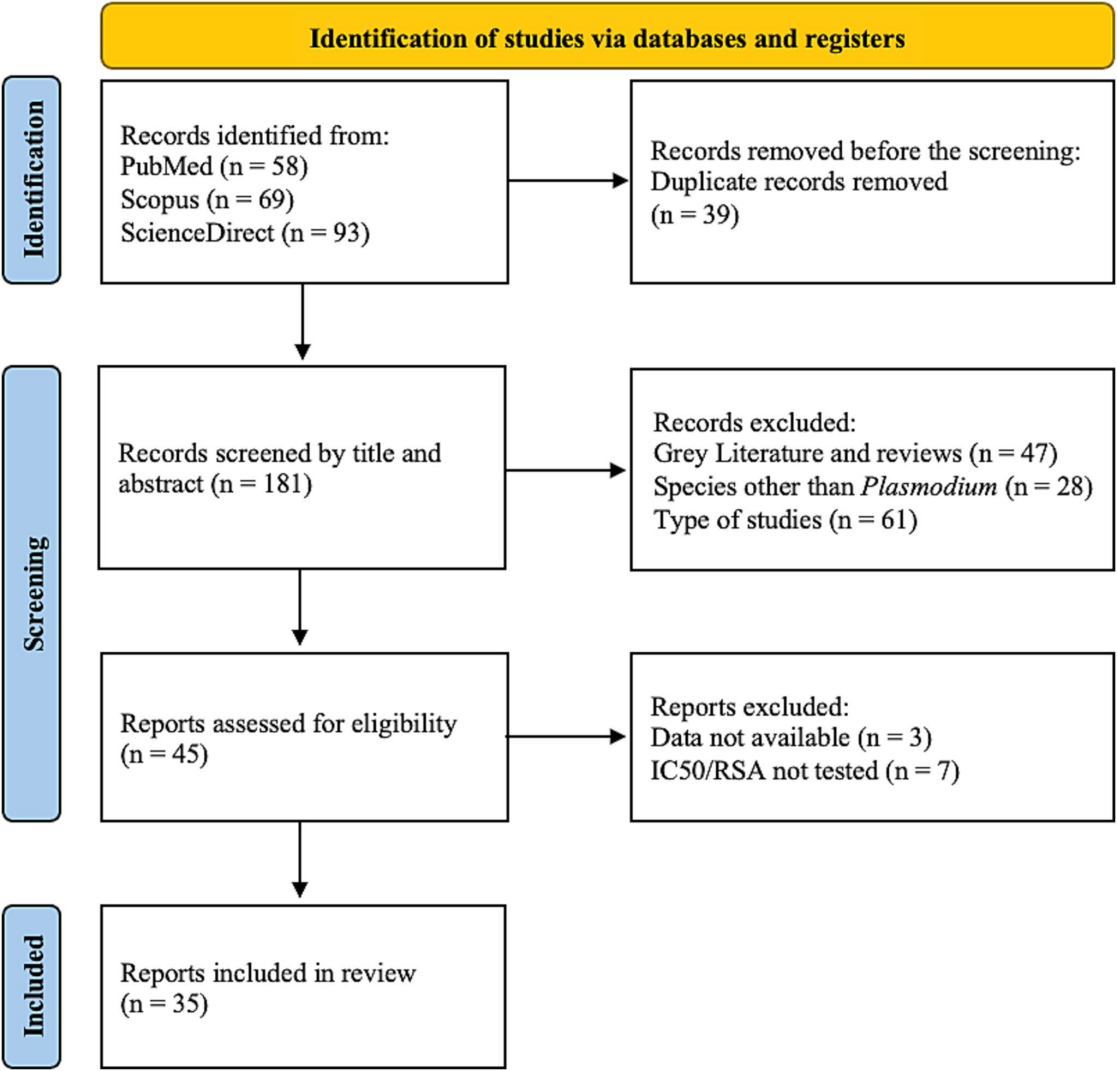
PRISMA 2020 flow diagram for study selection [adapted from (22)].

The literature search for this systematic review was conducted according to the PRISMA 2020 Statement (22). Figure 1 summarizes the phases of the search. A total of 220 articles were first retrieved. After removing 39 duplicates, the title and abstract were analyzed and 136 articles were removed, due to gray literature, reviews, and type of study. Of the remaining articles, 10 were excluded, as they did not comply with the inclusion criteria. Finally, selected articles were analyzed based on their full-text content, resulting in 35 articles assessed for qualitative assessment.

#### 3.1.1 Characteristics of included publications

Individual characteristics of the 35 publications included in the systematic review are summarized in Table 1, which comprises 19 articles regarding the development of new proteasome inhibitors to be used as partner drugs and overcome antimalarial resistance (13, 18, 21, 24–39), 7 articles involving mutations that confer differences to the parasite in response to antimalarials (4, 10, 15, 40–46), 6 articles that comprise both the development of new proteasome inhibitors and associated mutations that can confer resistance and / or susceptibility (16, 47–51) and 3 articles where knockdowns or knockouts were performed (44, 45, 52).

**Table 1:** Studies summarized.

Type of Study	Reference	Antimalarials and its targets	Species	Lines	Susceptibility	Proteasome Activity
UPSI	(37)	DUB inhibitor	*P. berghei* *P. falciparum*	*Pb*820 *Pf*3D7	Synergize with DHA	-
UPSI	(28)	Ubiquitin E3 ligase: JNJ 26854165 HLI 373 Nutlin 3	*P. falciparum*	*Pf*D6 (CQ-S) *Pf*W2 (CQ-R)	Block the parasites’ development at the trophozoite and schizont stages	-
UPSI	(36)	β5: BTZ ZL_3_B	*P. falciparum*	3D7 Hb3 W2 Dd2	Block blood-stage development before DNA synthesis in drug-sensitive and resistant parasites lines	-
UPSI	(26)	SPP: NITD731 LY-411575 (Z-LL)_2_	*P. falciparum*	Dd2 3D7	Impair the degradation of unstable proteins and inhibit proteolytic activity	-
UPSI	(39)	20S: TDI8414	*P. falciparum*	3D7	Synergize with DHA	-
GM	(42)	DHA OZ439	*P. falciparum*	K13^C580Y^ K13^C580Y^ β2^C31Y^ K13^C580Y^ β2^C31F^ K13^C580Y^ β5^A20S^	C31Y and C31F sensitized parasites to DHA, even in the presence of the K13^C580Y^ mutation. A20S hypersensitizes to WLW but does not change DHA susceptibility in parasites with the K13^C580Y^ mutation	-
GM	(15)	DHA BTZ	*P. falciparum*	*pB*104 (STAR-related lipid transfer)	DHA regulates the cellular response to unfolded proteins. BTZ regulates proteasome activation	-
GM	(40)	DHA	*P. falciparum*	Rpn2^E738K^	Increases parasite survival in the presence of DHA	Increased chymotrypsin-like activity and decreased accumulation of polyubiquitinated proteins in the presence of DHA
GM	(41)	DHA	*P. falciparum*	CAM3.II K13^WT^ Rpt4^E380*^ CAM3.II K13^WT^ Rpn6^E266K^ CAM3.II K13^WT^ Rpt5^G319S^ K13^C580Y^	RPT4^E380*^and Rpn6^E266K^ sensitized parasites to DHA. G319S sensitized parasites to DHA at the ring and trophozoite stage. When associated with K13^C580Y^, these three mutations are sensitive to WLL and DHA in the IC_50_, but RPT5^G319S^ is resistant to WLW and DHA in the RSA.	Antimalarial compounds that synergize with proteasome inhibitors perturb parasite proteostasis. Early parasite UPR signaling in response to DHA dictates eventual survival outcomes. Parasite susceptibility to DHA correlates with dysfunction in proteasome-mediated protein degradation.
UPSI GM	(50)	β5: AsnEDAs (WHZ-04)	*P. falciparum*	β6^A117D^ β5^A49S^	Synergize with DHA and with β2 inhibitors	-
GM	(44)	DHA Amodiaquine Piperaquine MFQ CQ	*P. falciparum*	*Pf*RFUL KD	Increased sensitivity to all, except CQ	Decreased ubiquitination
UPSI GM	(51)	β5: TDI8304	*P. falciparum*	3D7 Dd2 Dd2 β6^A117D^ Dd2 β5^A49S^ Hb3(CQ-S) 3,663 (ART-S) 4,884 (ART-R)	TDI8304 demonstrated comparable activity against all the strains, except in the Dd2 β6^A117D^. It synergizes with DHA in both sensitive and resistant parasites	Accumulation of polyubiquitinated proteins. TDI8304 blocked the labeling of β5 subunit dose-dependently, but not β1 or β2
UPSI GM	(49)	β2 and β5: WLL β2: WLW	*P. falciparum*	K13^R539T^ Rpt4^E380*^ Rpn6^E266K^ K13^C580Y^ β5^A20S^ K13^C580Y^ β2^C31Y^ β6^A117V^ β2^C31F^ K13^C580Y^ β6^S108L^ K13^C580Y^ β2^A49E^	After selection of mutants: modest gain of resistance Hypersensitivity to the other inhibitor in some mutations	-
UPSI	(29)	β5: AsNEDAs (PKS21004 and PKS21003)	*P. falciparum*	3D7 (SUL-R) Hb3 (PYR-R) D6 (PAN-S) Sb1-a6 (ATOV-R) Dd2 (MDR) V1S (MDR) 3,663 (ART-S) 5,188 (ART-S) 4,884 (ART-R) 5,202 (ART-R) 4,912 (ART-R)	Synergize with DHA and with β2 inhibitors	-
UPSI	(35)	20S: Salinosporamide A	*P. falciparum*	3D7	Inhibition of the 20S subunit. Protected mice against malaria infection	Accumulation of ubiquitinated proteins
GM	(43)	ART	*P. berghei*	K13^WT^ UBP-1^V2721F^ K13^F458I^ K13^M488I^ K13^Y505H^ K13^R551T^	Reduced susceptibility of K13^M488I^, K13^R551T^, K13^Y505H^, compared to the equal susceptibility in K13^WT^, UBP-1^V2721F^, and K13^F458I^	-
GM	(4)	DHA TDI8304	*P. falciparum*	K13^R539T^	Clones highly resistant to DHA have almost no change in EC_50_ values for TDI-8304	-
UPSI GM	(47)	β2 and β5: WLL EY 4–78 β2: WLW β5: J-50 J-71 J-78 J-80 WHZ-04 TDI4258 TDI8304 Compound 4 Compound 6 BTZ Epo	*P. falciparum*	Dd2^WT^ Cam 3.II K13^C580Y^ V1/S K13^C580Y^ Cam 3.II K13^C580Y^ β2^C31Y^ V1/S K13^WT^ β2^C31F^ V1/S K13^C580Y^ β2^A49E^ Cam 3.II K13^C580Y^ β5^A20S^ Dd2-B2 β5^A20V^ Dd2-B2 β5^M45I^ Dd2-B2 β5^A49S^ V1/S K13^WT^ β6^A117V^ Dd2-B2 β6^A117V^ V1/S K13^C580Y^ β6^S108L^ Rpn6^E266K^ Rpt5^G319S^ Rpt4^E380*^ Dd2 β5^M45R^ Dd2 β5^M45V^ Dd2 β5^A50V^ DD2 β6^N151Y^ DD2 β6^S157L^	Of all the compounds tested, the vinyl sulfone inhibitors, WLL, WLW, and EY 4–78 presented the smallest IC_50_ shift across the panel of mutant lines	-
UPSI GM	(16)	β5: Compound 27 8,304-*vs* β5 and β6: TDI8304	*P. falciparum*	Dd2-B2 β5^M45I^ Dd2-B2 β6^S157L^ Dd2-B2 β6^N151Y^	TDI-8304 and 8,304-*vs* induced selective and potent parasite inhibition. 8,304-*vs* showed a higher drop in potency in adapted parasites	-
UPSI GM	(48)	β2 and β5: WZ32 TDI4258 TDI8239 TDI8304 MMV1579506 MMV1581599 MMV1794229	*Ugandan P. falciparum isolates*	β2^S214F^ β2^I204T^ β5^A142S^ β5^D150E^ Rpn10^T225S^ Rpn10^E380L^ *Pf*3d7_0808300^M144I^	β2^S214F^ decreased susceptibility to MMV1579506 and MMV1794229. All the other showed no differences.	-
GM	(10)	Epo Carfilzomib BTZ ART	*P. falciparum*	K13^WT^ (PL2) K13^Y493H^ (PL1) K13^C580Y^ (PL5) K13^R539T^ (PL7)	Epo, carfilzomib, and BTZ synergize with DHA, in all parasite lines	-
UPSI	(30)	20S: Copper (II) complexes Zinc (II) complexes CQ ART	*P. falciparum*	*Pf*3D7 (ART-S) *Pf*5202 (ART-R)	Copper (II) complexes have antimalarial potency against CQ and ART-sensitive and resistant parasites. Zinc (II) complexes, only have antimalarial activity against sensitive parasites.	Only copper (II) complexes inhibited the β2 subunit of the proteasome in both strains
UPSI	(21)	β2: LLL LLW β2 and β5: WLL WLW	*P. falciparum*	PL2 (ART-S) PL7 (ART-R)	Synergy between DHA and WLW	WLL inhibits all 3 proteasome activities
UPSI	(38)	β2 and β5: PFS (PW28)	*P. falciparum* *P. berghei*	D10 (MDR) Dd2 (MDR) 3D7 (CQ-S) Clinical Isolates from Lambaréné	Active against sensitive and resistant parasites	PSF inhibits proteasomal activity by inhibiting β2 and β5 but not β1
UPSI	(25)	β5: Thiostrepton and Derivatives (SS231 and SS234) MG132	*P. falciparum*	3D7 (ART-S) Dd2 (ART-R)	Active against CQ-sensitive and -resistant parasites	Accumulation of ubiquitinated proteins in all, especially in Thiostrepton and SS234
UPSI	(27)	β5: GTX	*P. falciparum*	K-1 (CQ-S) FCR-3 (CQ-R)	Decreases proteasome activity	Decreased chymotrypsin-like activity
UPSI	(18)	USP14: b-AP15	*P. falciparum*	*Pf*USP14 3D7 (CQ-S) Dd2 (CQ-R)	Strong antimalarial activity against all strains	Accumulation of polyubiquitinylated substrates but proteasome activity unaltered
GM	(52)	DHA	*P. falciparum*	3D7 PA28 KO	Increased sensitivity to DHA in the KO	*Pf*PA28 enhances the cleavage of the fluorogenic substrate by *Pf*20S
UPSI	(13)	DHA CHX E1: C1 NEDD8: MLN4924	*P. falciparum*	3D7	CHX, C1, and MLN4924 antagonize with DHA	Decreased polyubiquitinated proteins and phosphorylation of eIF2α (key toxic events initiated by DHA treatment)
UPSI	(24)	β5: J-78 J-80	*P. falciparum*	Dd2	Both synergize with DHA and are highly selective for erythrocytic-stage parasites	J-78 has high potency for β2 and β5 subunits and high selectivity of J-80 for Pf20S β5 over c20S β5
UPSI	(34)	β5: GW012X	*P. falciparum*	Cam3.II 3D7	Enhances DHA activity	Inhibits proteasome-mediated degradation
UPSI	(32)	β5: Carmaphycin B Analog 18	*P. falciparum*	*Pf*Dd2	Synergize with DHA	β5 activity decreased
UPSI	(33)	β2 and β5: LU102 β5: PR709A	*P. falciparum*	D10	Synergize with DHA	LU102 inhibits β2 and PR709A inhibits β5
UPSI	(31)	β2 and β5: Copper (II) complexes	*P. falciparum*	*Pf*3D7 *Pf*5202 (ART-R)	Copper (II) complexes have antimalarial potency against CQ and ART-sensitive and resistant parasites	Copper (II) complexes 1,2 and 3 actively inhibit the proteasome
GM	(45)	ART E64 Lopinavir Nelfinavir Saquinavir Epo	*P. berghei* *P. falciparum*	*Pf*DDI1 KD	DDI1 KD increased parasite vulnerability and DHA susceptibility	DDI1 KD causes accumulation of ubiquitinated protein

UPSI, ubiquitin-proteasome system inhibitors; GM, Genetic Modifications. Characterization of the eligible studies regarding antimalarial studies, parasite species and lines (including mutations), susceptibility to the antimalarial and proteasome activity.

Concerning the *Plasmodium* spp., one study was performed in rodent malaria parasites *P. berghei*, three others were performed in both *P. berghei* and human malaria parasites *P. falciparum*, and the remaining were performed in *P. falciparum* parasites.

#### 3.1.2 Quality assessment

Each study quality was assessed using an adaptation for *in vitro* studies of the ARRIVE guidelines and it is summarized in Supplementary Table S1. A total of 14 criteria were evaluated, where a score between 0 and 1 was attributed to each criterion, and the mean score was calculated. Studies with a mean score higher than 12 were considered good, moderate for a score between 8.5 and 12, and weak for a score lower than 8.5. Of the 35 studies identified as relevant for this systematic review, 16 were rated as high (45.7%), 15 were rated as moderate (42.9%), and four as low (11.4%).

### 3.2 Development of UPS inhibitors

**Figure 2:**
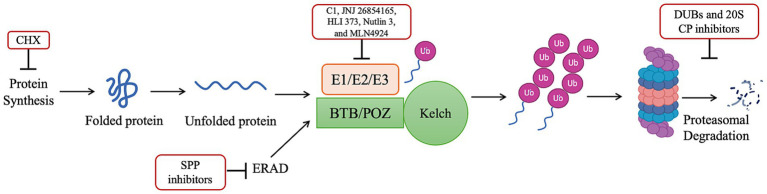
Ubiquitin-proteasome system and its inhibitors [adapted from (13)]. Ablation of protein synthesis through CHX and inhibition of the E1/E2/E3 ubiquitin machinery through C1, JNJ 26854165, HLI 373, Nutlin 3, and MLN4924, prevents the accumulation of polyubiquitinated proteins and enable cell survival. On the other hand, inhibition of the proteasome, through DUBs and 20S CP inhibitors causes accumulation of polyubiquitinated proteins and parasite killing. ERAD facilitates the degradation of misfolded proteins. However, the SPP inhibitors impair this facilitation and inhibit its proteolytic activity.

The development of new proteasome inhibitors for antimalarial partner drugs has grown in the last decade. In this systematic review, 25 articles report new compounds, of which 5 articles relate to inhibitors of the E1/E2/E3 ubiquitin machinery or ablation of protein synthesis, and 20 articles relate to the inhibition of the 20S core particle, responsible for the proteolysis (Figure 2). The first set of articles includes inhibitors of protein synthesis, the E1/E2/E3 machinery, deubiquitinating enzymes, and signal peptide peptidase (SPP), which targets endoplasmic reticulum-associated degradation (ERAD). The group of inhibitors of the 20S CP was mainly focused on the β2 and β5 subunits.

#### 3.2.1 Inhibitors of polyubiquitination

Different UPSI cause the accumulation of polyubiquitinated proteins, leading to parasite death. The protein synthesis can be inhibited by cycloheximide (CHX), by arresting protein translation, which antagonizes the action of dihydroartemisinin (DHA), since it reduces the DHA-mediated build-up of polyubiquitinated proteins (13).

The E1/E2/E3 machinery can be inhibited in different phases. Herein, there is an inhibitor of the E1, Compound 1 (C1: 5′-O-sulphamoyl-N(6)-[(1S) − 2,3-dihydro-1H-inden-1-yl]-adenosine), which depleted intracellular pools of activated ubiquitin, and, consequently, it antagonizes DHA killing (13); and three inhibitors of the E3: JNJ 26854165, HLI 373, and Nutlin 3. These last three block the development of *P. falciparum* parasites at the trophozoite and schizont stages (28). Additionally, an inhibitor of NEDD8, MLN4924, is suggested to involve a cullin E3-mediated ubiquitin ligation event and antagonizes DHA-mediated killing (13).

Six different DUBs inhibitors were studied: PR-619 (broad spectrum DUB inhibitor), P5091 (USP7 and USP47), TCID (UCH-L3 and UCH-L1), WP1130 (UCH-L1, USP9X, USP14, UCH37), b-AP15 (USP14 and UCH-L5) and NSC-632839 (USP2, USP7, SENP2). TCID, which is a highly selective mammalian UCH-L3 inhibitor, presented no activity against *Pb*820 and *Pf*3D7. This lack of activity can be attributed to structural differences between the mammalian and plasmodial enzymes. Nevertheless, the other five DUB inhibitors can impede intraerythrocytic development and can be combined for an additive effect. Inhibiting these upstream components of the UPS can enhance the activity of ART and effectively overcome ART resistance (18, 37).

Finally, inhibitors of SPP, targeting the ERAD pathway, such as (Z-LL)_2_, LY-411575, NITD679, and NITD731. The ERAD pathway is a cellular pathway that targets misfolded proteins of the endoplasmic reticulum for ubiquitination and subsequent degradation by the proteasome. Its inhibitors impair the protein’s ability to facilitate the degradation of unstable proteins and inhibit its proteolytic activity. These inhibitors have high selectivity and potency against *P. falciparum* parasites and are lethal to CQ-resistant parasites (26).

#### 3.2.2 Inhibitors of the 20S CP

Most articles discussing the development of new proteasome inhibitors focus on 20S CP inhibitors, which can be classified by the specific subunit they target. β2 inhibitors: peptide vinyl sulfones (LLW and WLW); peptide boronate (MMV1579506, MMV1581599, MMV1794229, and bortezomib (BTZ)). β5 inhibitors: asparagine ethylenediamine (AsnEDAs - WHZ-04, WZ32, TDI4258, PKS21004, and PKS21003); macrocyclic peptides (TDI8304, TDI8414 and TDI8239); epoxyketones (J-50, J-71, J-78, J-80, LU102, PR709A, carmaphycin B, and epoxomicin (Epo)); N, C-capped peptides; thiostrepton and derivatives (SS231 and SS234); gliotoxin (GTX); nonpeptidomimetic (GW012X). Both subunits, β2 and β5 inhibitors: peptide vinyl sulfones (WLL, LLL, and EY4-78); metal complexes (copper (II) and zinc (II) complexes); peptide sulfonyl fluorides (PFS - PW28); salinosporamide A.

Peptide vinyl sulfones are potent covalent and irreversible inhibitors, even against mutant parasites resistant to other proteasome inhibitors. They do not readily select for resistance, especially WLL, which binds to the catalytic β2 and β5 subunits, which is a key feature of these inhibitors. Cross-resistance between WLL and WLW was not observed. In mutations that confer resistance to one, there is increased susceptibility to the other. Additionally, this category of proteasome inhibitors synergizes with DHA in sensitive and resistant parasites (21, 47, 49).

Peptide boronates are covalent and slowly reversible inhibitors that demonstrated potent activity against *P. falciparum* isolates. BTZ is effective against drug-sensitive and resistant parasites (tested against four different strains of *P. falciparum* 3D7, Hb3, W2, and Dd2 that are either sensitive or have various levels of resistance to the antimalarial drugs pyrimethamine (PYR) and CQ) and blocks blood-stage development before DNA synthesis. However, it is a non-selective inhibitor, since it also binds to human proteasomes (36, 47, 48).

AsnEDAs are noncovalent and reversible inhibitors. They are active against erythrocytic, sexual, and liver-stage parasites and established laboratory strains of *P. falciparum* parasites with different drug-resistance profiles to ARTs, atovaquone (ATOV), PYR, and sulfadoxine (SUL). They synergize with DHA and with β2 inhibitors (29, 47, 48, 50).

Macrocyclic peptides, which present potent antiparasitic activity and low toxicity to human cells, and N, C-capped peptides have non-covalent and reversible inhibitory properties (16, 39, 47, 48, 51).

Epoxyketones are covalent and irreversible inhibitors. Epoxomicin has high activity against *P. falciparum* parasites, however, it is toxic to human cells. Therefore, analogs had to be encountered. Carmaphycin B is a potent covalent peptide-epoxyketone inhibitor from a marine cyanobacterium and synergizes with DHA. However, it is only selective for asexual blood stage *P. falciparum* parasites over human cells. Other analogs, such as J-78 and J-80, were identified, as being highly selective for erythrocytic-stage *P. falciparum* (24, 32, 33, 47).

Thiostrepton and derivatives and GTX, a fungal metabolite, are active against chloroquine (CQ)-sensitive and -resistant *P. falciparum* parasites. Thiostrepton rapidly eliminates parasites before DNA replication. It also arrests the schizont stage and acts on the gametocytes, accumulating ubiquitinated proteins in the erythrocytic stages (25). GTX decreases the chymotrypsin-like activity of the proteasome in a time-dependent manner and it has low levels of cytotoxicity (27).

Nonpeptidomimetic inhibitors are potent compounds, but they have low selectivity to *P. falciparum* proteasome. However, GW012X shows a fast-acting parasitological profile and strong synergy with DHA against artemisinin-resistant *P. falciparum* parasite (K13 mutant), acting at the early ring-stage (34).

Copper (II) complexes have antimalarial potency against both CQ and ART-sensitive and resistant parasites, unlike the zinc (II) complexes, which only demonstrate antimalarial activity against sensitive parasites. The latter causes no hemolysis of RBCs (red blood cells), while the first induced increased hemolysis in a concentration-dependent manner, as well as, induction of reactive oxygen species (ROS), 20S inhibition, loss of mitochondrial membrane potential, and morphological features indicative of apoptosis (30, 31).

PFS targets the plasmodial proteasome and acts on the erythrocytic stages and gametocytes, being active against multidrug-resistant (MDR) (D10 and Dd2) and CQ-sensitive (3D7) *P. falciparum* laboratory strains, as well as against *P. berghei* parasites. It demonstrated low cytotoxicity against human cells, but when tested *in vivo*, signs of toxicity were shown in mice (38).

Salinosporamide A, produced by a marine actinomycete, shows strong inhibitory activity against blood stages of the parasite cycle, inhibiting the 20S subunit. It was also demonstrated that it protected mice against malaria infection (35).

### 3.3 Genetic modifications

Regarding the articles with genetic modifications, a total of 16 studies were included in this systematic review, where 2 performed a knockdown (KD) of 2 different genes, *Pfrful*, and *Pfddi1*, 1 performed a knockout (KO) of the gene *PA28*, and 13 explored point mutations in 12 different genes: *β2*, *β5*, *β6*, *rpn2*, *rpn6*, *rpn10*, *rpt4*, *rpt5*, *k13*, *ubp-1*, ubiquitin regulatory protein (PF3D7_0808300) and StAR-related lipid transfer protein (PF3D7_0104200).

#### 3.3.1 Plasmodium falciparum ring finger ubiquitin ligase

The role of a *P. falciparum* ring finger ubiquitin ligase (*Pf*RFUL) in response to antimalarial drugs was investigated through a knockdown of the gene (44). The *Pfrful* gene is located on chromosome 10 and encodes a 1,129 amino acids protein with an E3 domain and a zinc finger (RING) domain. Lower levels of *Pf*RFUL protein resulted in a reduction in the ubiquitination of various parasite proteins. It also increased the parasite’s susceptibility to several drugs and modified two proteins crucial for parasite drug responses: decreased the protein levels of the *P. falciparum* multiple drug resistance 1 protein (*Pf*MDR1) and altered post-translational modifications, such as ubiquitination, in the *P. falciparum* chloroquine transporter (*Pf*CRT).

#### 3.3.2 Plasmodium DNA damage-inducible 1 protein

*Plasmodium* DNA damage-inducible 1 protein (DDI1) contains a UBL domain and a retroviral protease (RVP) domain. Some DDI1 proteins also contain a UBA domain. This protein is expressed across all the major life cycle stages, and it was shown that is important for parasite survival with the KD of the gene. Mice infected with *P. berghei* strains with DDI1 KD exhibited self-limiting infections and protected the recovered mice from subsequent infection, indicating the potential of these parasites as a comprehensive organism vaccine. *Pf*DDI1 is linked with chromatin and DNA-protein crosslinks. Its depletion increased vulnerability to DNA-damaging agents and led to an accumulation of DNA-protein crosslinks. It also increased susceptibility to the retroviral protease inhibitor lopinavir and antimalarial artemisinin (45).

#### 3.3.3 PA28

The proteasome is activated by binding protein regulators to the 20S CP, such as 19S RP and PA28, which was studied through its genetic deletion (KO) (52). The PA28 stimulates 20S peptidase activity in a ubiquitin and ATP-independent manner and its deletion increases sensitivity to DHA, suggesting a role in proteostasis. *Pf*PA28 and *Pf*20S have an asymmetric interface and there is conformational flexibility and a leaky interface between them, facilitating product egress from the proteasome under proteotoxic stress conditions and underlying the protective role of *Pf*PA28 against DHA-induced protein damage.

#### 3.3.4 β2

Five mutations were identified in the β2 subunit, responsible for the trypsin-like activity of the proteasome: C31F (42, 47–49), C31Y (42, 47–49), A49E (47, 49), S214F (42, 47–49), I204T (42, 47–49).

The first two mutations were identified as the most frequent in WLW-resistance, and both sensitized parasites to DHA, even in the presence of the K13 C580Y mutation, as well as to WLL. The A49E mutation was also identified in WLW resistance, but no sensitization to WLL was reported. The two last mutations, S214F and I204T, were identified in eastern Ugandan *P. falciparum* isolates, with 3 and 1 isolates, respectively. The I204T was not associated with differences in susceptibility to the tested compounds (TDI8304, MMV1579506, and MMV1794229), while the S214F presented decreased susceptibility to two peptide boronates (MMV1579506 and MMV1794229) but not against WLW and WLL.

#### 3.3.5 β5

In the β5 subunit, responsible for the chymotrypsin-like activity of the proteasome, it was found nine mutations: A20S (16, 42, 47–51), A20V (47), A49S (47, 50, 51), M45I (16, 47), M45R (47), M45V (47), A50V (47), A142S (42, 47–49), and D150E (42, 47–49).

The A20S mutation was identified in WLL-resistant parasites and conferred hypersensitization to WLW, but it did not change the susceptibility to DHA in parasites with the K13 C580Y mutation. The A20V and M45I mutations were selected using boronates (MMV1579506 and MPI-12, respectively) and both mutations exhibited high levels of resistance to epoxyketones (J-50, J-71, J-78, and J-80), as well as to WHZ-04 and TDI-4258. The M45I also showed resistance to BTZ. The A49S was selected with AsnEDA (TDI4258), and it was shown to be more resistant to PKS21004 (AsnEDA) and BTZ, but more susceptible to WLW and other AsnEDAs (WHZ-04, −12, −13). It did not change the susceptibility to TDI8304. The M45R and M45V were selected with J-80, which was shown to have both increased resistance to J-71, but different profiles regarding TDI8304, with M45R being hypersensitivity and M45V resistant, as well as in EY 4–78, where M45R presents no change and M45V is resistant. Th A50V was selected with J-71 and presented a low level of resistance to this compound, as well as to J-80, and TDI8304. The last two mutations, A142S and D150E, were isolated in eastern Ugandan *P. falciparum* parasites, being natural variants, with 4 and 1 isolates, respectively. Neither of the mutations was associated with altered susceptibilities to proteasome inhibitors (macrocyclic peptide (TDI8304) and AsnEDA (TDI4258)).

#### 3.3.6 β6

In this systematic review, five mutations were found in the β6 subunit of the proteasome, which has no proteolytic activity (29): A117D (47, 50, 51), A117V (47, 49), N151Y (47), S157L (47), and S208L (49).

The A117D mutation was selected with an AsnEDA (PKS21004), which confers resistance to AsnEDA (PKS21004 and TDI4258) but not to BTZ, carfilzomib and TDI8304. The A117V and S208L mutations were selected with WLL, with both demonstrating a minor increase of resistance to it, but only the A117V demonstrated a small increase in the resistance to WLW. Although N151Y and S157L were selected with TDI8304, the mutation S157L presented higher resistance to this compound. S157L also presented resistance to J-71, J-80, WLL, and EY 4–78, unlike N151Y.

#### 3.3.7 Rpn2

One mutation, E738K, was found in the 26S proteasome regulatory subunit *rpn2* gene of *P. chabaudi* parasites resistant to artesunate (AS) + mefloquine (MQ) (40). The rpn2 functions as a scaffold and coordinates the activity and placement of multiple ubiquitin-processing factors at the proteasome. The mutation was shown to increase parasite survival and chymotrypsin-like activity of the proteasome and decrease polyubiquitinated protein accumulation when subjected to DHA treatment in *P. falciparum* parasites.

#### 3.3.8 Rpn6

A single mutation was found in the rpn6 subunit, which is responsible for the AAA regulation, E266K, selected with WLW in K13^C580Y^ parasites (41, 47, 49). It increases the susceptibility of mutant parasites to WLL and DHA, but not to WLW.

#### 3.3.9 Rpn10

In this study, genes encoding proteasome subunits of *P. falciparum* isolates from eastern Uganda were sequenced (48). The rpn10 subunit within the 19S RP serves as a vital canonical ubiquitin receptor, targeting multiubiquitin chains. Through its C-terminal ubiquitin-interacting motif, this subunit identifies ubiquitinated substrates, facilitating their transport to the proteasome. Two mutations were found in this gene, E380L, and T225S, with 274 and 213 isolates, respectively, but these were not associated with altered susceptibility to proteasome inhibitors.

#### 3.3.10 Rpt4

Only one mutation was found in the rpt4 subunit, an unfoldase, E380*, selected with WLW in a K13^C580Y^ parasite line (41, 47, 49). E380* is susceptible to WLL and DHA, but not to WLW.

#### 3.3.11 Rpt5

One mutation was found in the rpt5 subunit, another unfoldase, located within the AAA domain, G319S. It was selected with WLW in a K13^C580Y^ parasite line (41, 47, 49). This mutation conferred an increase in the WLW IC_50_ levels compared to the parental line, while there was no significant change in the WLL IC_50_ levels. Regarding to DHA, in the RSA, mutant parasites remained resistant when compared with the WT line, but are partially sensitized regarding the K13^C580Y^ parental line.

#### 3.3.12 K13

Artemisinin resistance is frequently associated with the K13 protein. Herein, seven different mutations were studied: C580Y (10, 41, 42, 47, 49), F458I (43), M488I (43), R539T (4, 10, 49), R551T (43), Y493H (10), and Y505H (43).

C580Y, the most prevalent mutation in Southeast Asia conferring resistance to DHA, and R539T, which confers a high level of resistance *in vitro* to DHA, do not display altered susceptibility to proteasome inhibitors, WLL, and WLW. The 539 T mutation is susceptible to TDI8304. F458I, M488I, R551T, and Y505H mutations in *P. berghei* are the equivalent of the *P. falciparum* F446I (the most common in Southern China, near the Myanmar border), M476I, R539T, and Y493H (commonly observed in Cambodia) mutations, respectively. The F458I mutation does not confer any change in the susceptibility to DHA, unlike the other three that display reduced susceptibility in 24-h assays and increased survival in *P. berghei*-adapted RSAs. M488I and R551T mutations mimic the delayed parasite clearance phenotype *in vivo* upon AS treatment and achieve faster recrudescence than wild-type parasites at high DHA doses. In the C580Y, R539T, and Y493H mutations, it was demonstrated that epoxomicin, carfilzomib, and bortezomib synergize with DHA, especially in the early ring stage.

#### 3.3.13 UBP-1

A single mutation, V2721F, in a ubiquitin hydrolase, UBP-1, was found in *P. berghei* parasites (43). The mutant parasite displays equal sensitivity to DHA as the WT when using the standard assays to *P. falciparum*, IC_50_, and RSA (*in vitro* standard and adapted to *P. berghei* assays). This mutation increased to 70% in the population compared to the WT line under CQ treatment, confirming that it provides a survival advantage.

#### 3.3.14 Ubiquitin regulatory protein (PF3D7_0808300)

One mutation, M144I, was found in a ubiquitin regulatory protein (PF3D7_08083000) (48). This mutation was identified in 141 isolates of 627 from eastern Ugandan. However, there were no alterations in susceptibility to any of the tested compounds (WZ32, TDI4258, TDI8239, TDI8304, MMV1579506, MMV1581599, and MMV1794229).

#### 3.3.15 StAR-related lipid transfer protein (PF3D7_0104200)

This study demonstrated that mutations in the StAR-related lipid transfer protein (PF3D7_0104200) conferred increased susceptibility to both DHA and BTZ (15). It was demonstrated that distinct modulation of specific exportome- and organelle-linked lipid metabolism components play a vital role in differentiating the response of *P. falciparum* to artemisinin compared to the UPS. DHA regulates the cellular response to unfolded proteins, while BTZ regulates proteasome activation by downregulating proteins involved in proteasomal catabolic processes and upregulating the PA28 activator.

## 4 Discussion

Malaria remains a major health threat worldwide. The emergence of partial resistance to ARTs and to the partner drugs used in ACTs is especially alarming, given their widespread use in endemic populations. Therefore, there is an urgent need to develop drugs with novel mechanisms of action and broad therapeutic potential to enhance interventions and overcome multidrug resistance. The *Plasmodium* proteasome, crucial throughout the parasite’s life cycle, has garnered attention as an attractive target for new drug development (16).

In this systematic review, 35 articles were extensively examined for the proteasome role in antimalarial resistance. The results of this systematic review were categorized into two groups of articles: those focused on the development of UPS inhibitors and those addressing genetic modifications that enhanced the understanding of the proteasome’s role in antimalarial response and resistance. Concerning the first category, 25 articles were evaluated, identifying six proteasome components targeted by inhibitors: protein synthesis, DUBs, E1/E2/E3 machinery, ERAD pathway, and β2 and β5 subunits of the 20S CP. This resulted in a total of 16 categories of inhibitors, with 12 related to the 20S CP subunits. Inhibition of the protein synthesis and the E1/E2/E3 machinery antagonizes ARTs activity. However, apart from the ERAD pathway inhibitor, for which there is no available information, all other targeted subunits, which are all downstream of the UPS, can synergize with ART and potentially serve as partner drugs in ACTs. This demonstrates that defective presentation of proteins for ubiquitination may allow cell survival in ART-resistant parasites. Therefore, targeting DUBs or proteasome components can be a means of overcoming resistance-inducing mutations.

As mentioned, 20S CP subunits are the most targeted, mainly those responsible for the trypsin-like and chymotrypsin-like activity of the proteasome. *P. falciparum* parasites exhibit sensitivity to short-term inhibition of the β5 subunit, especially during parasite schizogony. Furthermore, simultaneous inhibition of the β2 and β5 subunits leads to efficient parasite elimination at all stages of the asexual form of *P. falciparum* (33). WLL was identified as having the most favorable profile, with a low risk of selecting for resistance and sustained potency against a panel of proteasome mutants, which is attributed to the covalent nature of this inhibitor and its irreversible dual binding to the β2 and β5 subunits (47). Regardless, none of these compounds are in advanced phases of development (53). According to the Malaria Drug Accelerator (MalDA),^1^ a consortium of 15 leading scientific laboratories, the majority of the 20S CP inhibitors are in the third class of validated antimalarial target (VT3), having: 1. Genetically validated target (gene encoding the target demonstrates essentiality from genetic modification assays); 2. Chemically validated target (inhibition demonstrated with tool compounds); 3. All of the above criteria plus a known resistance potential (which can be determined with known mutation and/or gene amplifications in WT parasites from *in vitro* evolution assays) (54).

A significant hurdle to advancing drug development efforts is the substantial host toxicity induced by proteasome inhibitors that also target the mammalian proteasome. Additionally, it is important to identify compounds with low resistance development risks early in the drug development process, such as peptide vinyl sulfones, which present a low propensity for resistance selection *in vitro* (47), as well as use proteasome inhibitors combination therapies to avoid the easy development of resistance. Developing the next generation of antimalarial drug combinations with the durability to resist selective pressures must be a top priority in the fight against malaria and other global infectious diseases (55).

In the genetic modifications category, 16 articles were reviewed. These articles collectively studied a total of 15 genes undergoing genetic modifications, which could involve knockdowns, knockouts, or mutations. Two genes were subjected to knockdown to investigate the proteasome response to antimalarials, while one gene underwent knockout. Herein, a total of 35 mutations were assessed, with 28 resulting in alterations in susceptibility to either ARTs, proteasome inhibitors, or both. The 20S CP was the most prone to mutations, since it accounted for the majority, 19 mutations, followed by the K13, accounting for 9 mutations, and the 19S RP, which accounts for 6 mutations.

Artemisinin resistance is frequently associated with K13 mutations. However, the exact mechanism by which it confers resistance remains unclear. One hypothesis was the maintenance of the UPS when subjected to ART since this antimalarial is thought to inhibit proteasome degradation. However, synergy between ART and proteasome inhibitors, in particular WLL and WLW, was observed in both K13 mutant and WT parasites, demonstrating that these mutations do not modulate proteasome activity (10, 41, 42). One alternative hypothesis is that K13 mutant parasites have reduced hemoglobin uptake and digestion and, consequently, decreased artemisinin activation. WT parasites have hyperactivated unfolded protein response (UPR) when subjected to DHA, and mutant parasites present lower UPR activation in ring-stage parasites, being consistent with the last hypothesis (12, 56, 57). In the late stages, K13 is not involved in hemoglobin uptake, but the UPR activation is earlier in DHA-treated K13 mutant trophozoite parasites. Therefore, although K13 does not mediate artemisinin resistance by modulating proteasome activity, it can modulate UPR activation and resolution (10, 41, 42).

Mutations in the 26 s proteasome confer distinct patterns of resistance to different inhibitor classes. Mutants showing resistance to proteasome inhibitors were shown to exhibit increased vulnerability to ART. Even a strain with dual mutations in both the proteasome and K13 demonstrated higher susceptibility to DHA, suggesting that resistance to ART and proteasome inhibitors seem mutually exclusive. Additionally, proteasome mutations that conferred resistance to one inhibitor, conferred hypersensitivity to another, creating a potential for resistance-refractory inhibitor combinations. These highlights make the combination of ARTs and 20S inhibitors an attractive prospect for the development of antimalarial treatment regimens (4, 47).

Nevertheless, this systematic review has some limitations. A major challenge was the diversity of phenotypic testing methods, especially in the assessment of the proteasome activity. For this reason, all the studies with susceptibility assessed with IC_50_ were included, even though this method does not correlate entirely with *in vivo* artemisinin resistance phenotype.

As previously mentioned, *P. falciparum* and *P. vivax* are the most common parasites causing infections in humans. *P. vivax* was long considered non-lethal, but this perception has gradually shifted with the rising incidence of severe cases and fatalities associated with the parasite (58). Therefore, *P. vivax* research has been hindered by several factors, including inaccurate diagnosis, the parasite’s ability to form a dormant liver stage, early transmission, and technical challenges in establishing continuous *in vitro* cultures. Nowadays, the first-line treatment for *P. vivax* is still CQ and recently ACTs. Nevertheless, it is important to encounter new alternatives. One study focused on the *P. vivax* proteasome, specifically investigating the putative circumsporozoite protein (*Pv*puCSP). *Pv*puCSP plays a crucial role in regulating protein degradation in *P. vivax* and presents a promising target for drug development (1). Although *P. knowlesi* can serve as a model for *P. vivax* infections, there is still limited information available about the proteasome in this species as well (59). It is of high interest to address this knowledge gap.

In summary, this systematic review discusses available UPSI, their respective highlights and limitations, mutations within the UPS, and their phenotypic impact on antimalarial response. With this, we aim to enhance the understanding of the proteasome function in the malaria parasite lifecycle and its role in antimalarial response, particularly concerning resistance, an increasingly critical factor threatening the effectiveness of current treatments. Additionally, we endeavor to propel the development of more effective therapies, showcasing proteasome inhibitors as ideal candidates for adjunctive use in ACTs, addressing ART-resistant malaria.

## Data Availability

The original contributions presented in the study are included in the article/Supplementary material, further inquiries can be directed to the corresponding authors.

## Footnotes

^1^https://www.malariada.org/
