# Supplementary material for: Ubiquitin-proteasome system in Plasmodium: a potential antimalarial target to overcome resistance – a systematic review
Source: Front Med (Lausanne). 2024 Oct 21;11:1441352. doi: 10.3389/fmed.2024.1441352 (PMC11532105; doi:10.3389/fmed.2024.1441352)
Supplement: Supplementary file 1 [file Table_1.DOCX]

Supplementary Material

**Proteasome in *Plasmodium*: Deciphering its role for antimalarial resistance – a systematic review.**

Adriana F. Gonçalves^1,2^; Ana Lima-Pinheiro^1,2^; Pedro E. Ferreira^1,2^

^1^Life and Health Sciences Research Institute (ICVS), School of Medicine, University of Minho. Gualtar, 4710-057, Braga, Portugal

^2^Life and Health Sciences Research Institute (ICVS)/ Biomaterials, Biodegradables and Biomimetics Research Group (3B's)-PT Government Associate Laboratory, 4710-057 Braga, Portugal

*** Correspondence:**Adriana F. Gonçalves (id10896@alunos.uminho.pt)
Pedro E. Ferreira ([pedroferreira@med.uminho.pt](mailto:pedroferreira@med.uminho.pt))

# Supplementary Tables

**Table S1. Quality assessment of eligible studies.** This quality assessment was performed using an adaptation of th ARRIVE (Animal Research: Reporting of *In Vivo* Experiments) guidelines (Percie du Sert et al., 2020). 14 criteria were evaluated: 1) study design, (2) inclusion and exclusion criteria, (3) outcome measures, (4) statistical methods, (5) description of species, (6) experimental procedures, (7) results, (8) abstract, (9) background, (10) objectives, (11) interpretation and/or scientific implications, (12) generalizability/translation, (13) data access and (14) declaration of interests.
